# The Role of Gene Therapy and RNA-Based Therapeutic Strategies in Diabetes

**DOI:** 10.3390/ijms262110264

**Published:** 2025-10-22

**Authors:** Mustafa Tariq Khan, Reem Emad Al-Dhaleai, Sarah M. Alayadhi, Zainab Alhalwachi, Alexandra E. Butler

**Affiliations:** 1 School of Medicine, Royal College of Surgeons In Ireland-Medical University of Bahrain, Busaiteen P.O. Box 15503, Bahrain; 23200486@rcsi-mub.com (M.T.K.); 22200026@rcsi-mub.com (R.E.A.-D.); 22200394@rcsi-mub.com (S.M.A.); 2 Research Department, Royal College of Surgeons In Ireland-Medical University of Bahrain, Busaiteen P.O. Box 15503, Bahrain; zainab.halwachi@outlook.com

**Keywords:** gene therapy, RNA (ribonucleic acid) therapeutics, Type 1 diabetes, Type 2 diabetes, clustered regularly interspaced short palindromic repeats/CRISPR-associated protein 9 (CRISPR/Cas9)

## Abstract

Gene therapy and RNA (ribonucleic acid)-based therapeutic strategies have emerged as promising alternatives to conventional diabetes treatments, significantly expanding the therapeutic landscape using viral and non-viral vectors, and RNA modalities such as mRNA (messenger ribonucleic acid), siRNA (small interfering ribonucleic acid) and miRNA (micro ribonucleic acid). Recent advancements in these fields have led to notable preclinical successes and ongoing clinical trials, yet they are accompanied by debates over safety, efficacy and ethical considerations that underscore the complexity of clinical translation. This review offers a comprehensive analysis of the underlying mechanisms by which these treatments target diabetes, critically evaluating the fundamental concepts and mechanistic insights that form their basis, while highlighting current research gaps, such as the challenges in long-term stability and efficient delivery of RNA-based therapies, and potential adverse effects associated with gene therapy techniques. By synthesizing diverse perspectives and controversies, the review outlines future directions and interdisciplinary approaches aimed at overcoming existing hurdles, ultimately setting the stage for innovative, personalized diabetes management and addressing the broader clinical and regulatory implications of these emerging therapeutic strategies.

## 1. Introduction

Gene therapy and ribonucleic acid (RNA)-based therapeutics represent transformative strategies poised to address the growing global burden of diabetes, a condition that is projected to affect 643 million by 2030 and 783 million by 2045 [1]. Despite advancements in insulin formulation, delivery devices and glucose monitoring, most patients with Type 1 (T1D) and Type 2 (T2D) diabetes remain dependent on exogenous insulin or small-molecule drugs that treat symptoms rather than the underlying cellular and genetic dysregulations [2].

By leveraging viral and non-viral vectors to deliver therapeutic genes, gene therapy offers the potential for durable correction of β-cell deficiencies or enhancement of peripheral insulin sensitivity. Recent narrative reviews highlight successful preclinical applications, such as using adeno-associated virus (AAV) and lentiviral platforms to restore insulin production, protect β-cells from autoimmune attack and modulate glucose homeostasis in both rodent and large-animal models [3]. Complementing these approaches, RNA modalities, including messenger ribonucleic acid (mRNA), small interfering ribonucleic acid (siRNA) and micro ribonucleic acid (miRNA), enable transient, tunable regulation of gene expression to fine-tune metabolic pathways and immune responses. A growing body of work demonstrates that lipid nanoparticle-encapsulated mRNAs can induce therapeutic protein expression in target tissues, while chemically modified siRNAs and miRNA mimics achieve potent gene silencing with minimal immunogenicity [4]. Thus, gene addition offers potential permanence while RNA modalities provide temporal control, a complementary paradigm that frames subsequent sections.

However, both gene and RNA therapeutics face critical challenges in delivery, specificity, safety and scalability. For gene therapy, integrating viral vectors carries a risk of insertional mutagenesis; for example, five X-linked severe combined immunodeficiency (SCID-X1) patients successfully treated with ex vivo retroviral interleukin 2 receptor subunit gamma (*IL2RG*) gene therapy developed T-cell leukemia, and four harbored insertional mutations at *LMO2*, a known T-cell oncogene [5]; furthermore, pre-existing neutralizing antibodies against AAV capsids occur in approximately 30–60% of individuals, significantly reducing transduction efficiency [6]. Conversely, RNA platforms contend with rapid extracellular degradation, endosomal escape barriers and cold-chain logistics, as one of the foremost limitations of mRNA–LNP (lipid nanoparticle) therapeutics is their poor ability to breach endosomal membranes and enter the cytosol, thus preventing the encoded mRNA from accessing the translational machinery [6]. Moreover, ethical and regulatory frameworks for long-term surveillance and equitable access remain under development. Addressing these scientific and societal barriers will require coordinated advances in vector engineering, delivery platforms, regulatory policy and equitable implementation.

In this review, we first dissect the fundamental mechanisms underpinning gene and RNA interventions in diabetes, then evaluate mechanistic insights and therapeutic modalities across viral, non-viral and RNA platforms. We next explore convergent strategies that combine gene and RNA therapies for synergistic metabolic control, before examining societal integration considerations. By synthesizing current advances, controversies and translational barriers, we aim to chart a path forward for individualized, durable and widely accessible genetic and epigenetic therapies in diabetes care.

## 2. Fundamental Concepts and Mechanisms

Gene therapy represents a promising approach for addressing the underlying genetic and cellular dysfunctions in both T1D and T2D. In T1D, the autoimmune destruction of pancreatic β-cells leads to insulin deficiency. Gene therapy strategies aim to restore insulin production by promoting β-cell regeneration or protecting existing cells from immune-mediated damage. Approaches focusing on inducing new islets or modulating immune responses have been explored to re-establish endogenous insulin production; for example, localized expression of immunomodulatory factors such as insulin-like growth factor 1 (*IGF-1*) in pancreatic islets has been shown to preserve β-cell mass and counteract autoimmunity in non-obese diabetic (NOD) mice, highlighting the potential of combining β-cell protection with insulin restoration to achieve durable normoglycemia in T1D models [7]. However, challenges persist in replicating the precise glucose-responsive insulin secretion of native β-cells [8].

By comparison, T2D is characterized by insulin resistance and β-cell dysfunction and loss. Gene therapy efforts concentrate on enhancing insulin sensitivity and preserving β-cell function [9]. Targeting genes involved in glucose metabolism and insulin signaling pathways could yield improvement in glycemic control in T2D patients. For instance, studies have demonstrated that insulin signaling defects in skeletal muscle from T2D patients are characterized by impaired phosphorylation of insulin receptor substrate (*IRS*)-1, reduced phosphatidylinositol (PI) 3-kinase activity, and decreased glucose transport activity [10]. These defects contribute to reduced glucose uptake, indicating the importance of targeting these pathways in gene therapy approaches for T2D [11]. Although the underlying etiologies diverge in T1D and T2D, both have in common impaired β-cell function and disrupted insulin action, making shared therapeutic targets attractive. Ultimately, T1D and T2D differ greatly in many aspects, among them pathophysiology, typical age at onset, and treatment strategies (Table 1).

Similarly, overexpression of peroxisome proliferator-activated receptor gamma coactivator-1 alpha (PGC-1α) in skeletal muscle has been investigated to improve metabolic capacity; while early transgenic models showed enhanced mitochondrial biogenesis and fatty acid oxidation, they paradoxically reduced glucose transporter type 4 (GLUT4) expression and impaired insulin sensitivity. However, subsequent studies using moderate increases in PGC-1α demonstrated restoration of GLUT4 levels and improved insulin-stimulated PI3K/Akt activity, leading to significantly enhanced glucose uptake in rodent models of diabetes, though precise membrane trafficking dynamics warrant further investigation [12,13]. Consequently, any gene-based intervention targeting metabolic regulators must be coupled with delivery systems that permit controlled, tissue-restricted expression to avoid maladaptive effects. These studies demonstrate that PGC-1α gene therapy holds promise for correcting metabolic dysfunction in diabetes, but only when expression is carefully calibrated. This demonstrates the importance of gene therapy as a promising diabetes treatment method, aiming to restore insulin production, improve β-cell function and modulate glucose metabolism, with numerous preclinical and clinical studies proving its efficacy.

Gene therapy targets various tissues involved in glucose regulation. The primary focus is on restoring or protecting pancreatic β-cell function to re-establish endogenous insulin production. Recent strategies involve delivering key transcription factors such as Pancreatic and duodenal homobox 1 (*PDX1*), musculoaponeurotic fibrosarcoma bZIP transcription factor A (*MAFA*), and Paired box 6 (*PAX6*) to enhance β-cell function and survival, offering a personalized approach to diabetes treatment PDX1 [9]. The liver, as a central organ in glucose metabolism, can also be targeted to enhance insulin sensitivity and regulate glucose production [14]. Gene therapy approaches have included transferring insulin-producing genes into hepatocytes, enabling the liver to produce insulin in response to glucose, thereby improving glycemic control hepatocytes [15].

Additionally, modifying muscle and adipose tissues can improve glucose uptake and utilization, helping in overall glycemic control [16]. For instance, adiponectin gene therapy delivered to skeletal muscle has been shown to enhance insulin sensitivity and glucose homeostasis in diabetic models adiponectin [17]. Similarly, systemic gene delivery targeting adipose tissue has demonstrated improved glucose tolerance and metabolic health in preclinical studies [16]. Novel approaches can focus on reprogramming pancreatic ductal cells into insulin-secreting cells by delivering combinations of *PDX1* and *MAFA* via AAV vectors [18], effectively creating a renewable source of β-like cells within the native pancreatic microenvironment. Additionally, leveraging liver-specific promoters to achieve glucose-responsive transgene expression has minimized the risk of hypoglycemia while maintaining therapeutic insulin levels in T2D mouse models [19]. Therefore, development of gene delivery platforms capable of precise control over transgene dose, tissue specificity, and expression duration to maximize therapeutic benefit while avoiding off-target metabolic disturbances is warranted.

## 3. Gene Delivery Vectors

Effective gene therapy requires efficient delivery systems to introduce therapeutic genes into target cells to achieve therapeutic effects. To achieve these goals, different vectors are utilized to carry out specific gene modulation, including viral and non-viral vectors. Viral vectors, such as lentiviruses and AAVs, are commonly used due to their effective gene delivery capabilities. Lentiviral vectors have been utilized to deliver insulin genes specifically to pancreatic β-cells, resulting in improved glucose tolerance in diabetic models [20]. AAVs offer a favorable safety profile and have been applied for gene delivery to pancreatic islets [21]. Recent advances in AAV engineering, such as liver-specific Tet-off regulatable systems (an AAV8-based single-vector construct in which a liver-specific promoter (like albumin) drives the tetracycline-controlled transactivator (tTA), which, when doxycycline is not present, binds to tetracycline-responsive elements (TRE) to activate gene expression; when doxycycline is present, however, tTA is inhibited from binding, thereby turning off transgene expression in hepatocytes) provide a safety mechanism to turn off transgene expression, thereby mitigating risks of hypoglycemia associated with constitutive insulin secretion in vivo [22]. Moreover, novel serotype modifications (e.g., AAV8) with enhanced tropism for pancreatic tissues are under investigation to improve targeting specificity and reduce off-target integration [18]. Building on these insights, adenovirus-mediated overexpression of the transcriptional coactivator peroxisome proliferator-activated receptor gamma coactivator 1alpha (PGC-1α) in skeletal muscle has been shown to restore glucose transporter type 4 (GLUT4) expression and enhance insulin-stimulated insulin receptor substrate 1 (IRS-1) tyrosine phosphorylation and phosphoinositide 3-kinase (PI3K) activity, resulting in a threefold increase in glucose transport in diabetic rodent models; however, most of the protein is moved to the plasma membrane even in the absence of insulin [23]. In diabetic mice, glucagon-like peptide-1 (GLP-1) gene-delivering lentiviral vectors have enhanced insulin secretion and glucose tolerance [24]. Furthermore, there is significant promise in preclinical research using a broad range of animal models. In diabetic humanized liver mice, AAV2 vectors which contained the *PDX1* gene have improved hyperglycemia, indicating possible effectiveness in human hepatocytes [18]. These treatments are currently being translated into clinical settings. For example, KT-A112, an AAV-mediated gene therapy that targets skeletal muscle to deliver the genes for insulin and glucokinase, has demonstrated favorable outcomes in preclinical research and is moving closer to human clinical trials [25].

Non-viral vectors, including plasmid deoxyribonucleic acid (DNA) and lipid-based nanoparticles, present lower immunogenicity and are easier to produce. Plasmid-based gene therapy has demonstrated potential in restoring glucose homeostasis and promoting islet cell survival. Notably, plasmid-based approaches raise fewer safety concerns, as they are less likely to provoke an immune response due to the absence of viral components. This allows for repeated administration without any adverse reaction to the vector itself, enhancing the feasibility of long-term gene therapy strategies [26]. Furthermore, plasmid-based systems have been used to deliver genes encoding therapeutic proteins, such as GLP-1, resulting in sustained expression and improved glycemic control in diabetic animal models. For instance, chitosan/plasmid-DNA nanocomplexes have been shown to effectively deliver GLP-1 genes [27], leading to increased insulin secretion and decreased blood glucose levels for up to 24 days post-treatment [26]. Selection of an appropriate vector therefore follows directly from the intended biological target, desired expression kinetics and acceptable safety profile to undertake genetic modulation.

Another approach is gene addition, which involves introducing functional genes to compensate for defective or missing ones. One innovative approach involves introducing the insulin gene into non-β-cells, such as hepatocytes or fibroblasts, to restore insulin production. This strategy aims to create surrogate insulin-producing cells that are not targeted by the autoimmune response characteristic of T1D. However, challenges persist, including the need to equip these cells with glucose-sensing capabilities and the cellular components necessary for proper insulin processing and secretion [28,29]. For example, hepatotropic AAV8 vectors carrying optimized human insulin transgenes under glucose-responsive promoters reduced hyperglycemia in streptozotocin (STZ)-induced diabetic mice with improved dose–response kinetics, highlighting the importance of promoter design in controlling transgene expression (Table 2) [19].

Another mechanism of genetic modulation is correction of mutations affecting insulin production; one such method being clustered regularly interspaced short palindromic repeats/CRISPR-associated protein 9 (CRISPR/Cas9), which works by designing a single guide RNA (sgRNA) that directs the Cas9 enzyme to a specific DNA sequence [30]. The Cas9 enzyme then introduces a double-strand break in the DNA at the target site. The cell’s repair machinery attempts to fix this break, leading to gene disruption or, if a donor DNA template is provided, precise gene correction through homology-directed repair (HDR) [31]. Recent studies using AAV-CRISPR/Cas9 have successfully corrected mutations in the insulin receptor gene in inducible knockout mice, improving insulin sensitivity and glycemic control, which underscores the translational potential of in vivo gene editing for T2D-related genetic defects [32]. Furthermore, research shows that CRISPR technology can fix genetic abnormalities and regulate important metabolic pathways associated in the pathophysiology of diabetes, making it a promising therapeutic option [33]. Additionally, studies demonstrate that CRISPR can be used to modify immune components and stem cells, providing new approaches for immune regulation and β-cell replacement in both T1D and T2D [31]. Clinical translation of CRISPR-based therapies is being facilitated by the development of high-fidelity Cas9 variants that minimize off-target cleavage, thereby addressing safety concerns in gene correction applications for monogenic forms of diabetes such as Maturity-Onset Diabetes of the Young (MODY) and neonatal diabetes [34].

In addition to gene addition and correction of mutations, gene silencing, using RNA-based approaches, is another way to regulate genes. Although gene therapy has demonstrated potential in altering cellular and genetic processes to treat diabetes, RNA-based therapeutic approaches have quickly emerged as an alternative because they enable transient, programmable protein expression without entering or integrating into the host genome, are relatively simple and scalable to manufacture, and can be directed towards pathways that are difficult to target with small molecules [35,36,37]. RNA-based therapies for diabetes use a variety of RNA molecules (mRNA, siRNA and miRNA) in order to manage disease-related pathways, restore glycemic control and prevent complications.

**Figure 1:**
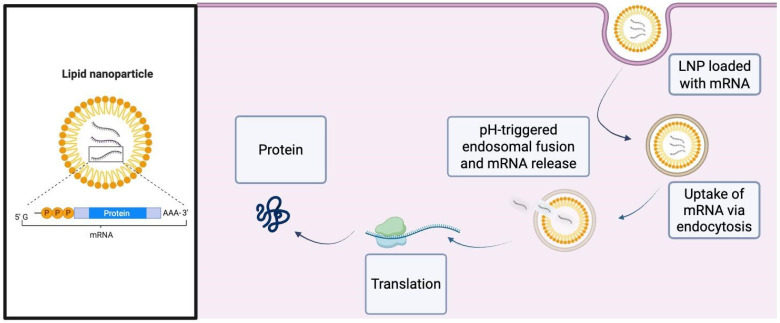
A simplified schematic illustrating how encapsulation of mRNA in lipid nanoparticles protects the payload from enzymatic degradation and immune detection, enhances cellular internalization, triggers pH-dependent lipid protonation for endosomal escape, and ultimately permits robust cytosolic translation of the encoded therapeutic protein. Abbreviations: LNP, Lipid Nanoparticle; mRNA, messenger ribonucleic acid; pH, potential of Hydrogen.

Exogenous mRNA encoded proteins like insulin, β-cell transcription factors or immunomodulatory drugs are introduced by mRNA therapies [38]. Lipid nanoparticles (LNPs), which encapsulate these structures, shield mRNA from damage and facilitate effective cellular uptake. Specifically, the LNPs encapsulate mRNA and protect it during circulation until they are internalized by target cells via endocytic pathways and become entrapped in endosomes. Within the acidifying endosome, the ionizable lipids protonate, promoting lipid–membrane interactions that destabilize the endosomal membrane and enable transient pores or fusion events through which a fraction of the mRNA escapes into the cytosol. Once released into the cytosol, chemically optimized mRNA is translated by ribosomes into the encoded protein (Figure 1). However, for LNPs to be able to function in the human body there are several barriers that need to be overcome, such as extracellular degradation; evasion of the mononuclear phagocyte system (MPS) and avoidance of renal clearance; reaching the target system; and finally, evasion from endosomes [39]. While staying neutral at physiological pH helps minimize toxicity and immunogenicity, ionizable lipids within LNPs become positively charged in acidic environments like those in the endosome, promoting membrane fusion and endosomal escape [40,41]. A recent study showed that by changing macrophage polarization toward the anti-inflammatory M2 phenotype, a reactive oxygen species (ROS)-responsive LNP-mRNA expressing interleukin 4 (IL-4) optimized wound healing was achieved in diabetic mice (Table 2) [41]. Furthermore, by inducing immunological tolerance, LNP-mRNA encoding autoantigenic epitopes of glutamic acid decarboxylase prevented the development of T1D in a non-obese diabetic (NOD) mice model (Table 2) [42]. mRNA-based strategies have advanced into pre-clinical models, focusing on peptide hormones relevant to diabetes. A recent study in nonhuman primates demonstrated that a single subcutaneous dose of lipid nanoparticle–encapsulated GLP-1 receptor agonist mRNA significantly reduced fasting glucose and appetite over 56 days, suggesting long-acting metabolic modulation [43]. Ionizable lipid nanoparticles employed to deliver mRNA for adiponectin in rodents improved insulin sensitivity and reduced hepatic fat buildup, suggesting a promising therapeutic route for T2D [44].

Despite much progress, important issues like dosing, release timing and long-term effectiveness still need to be established. Human clinical trials are being planned, with companies like Moderna developing GLP-1 mRNA therapies for T2D, although full trial details have not yet been released [44].

**Figure 2:**
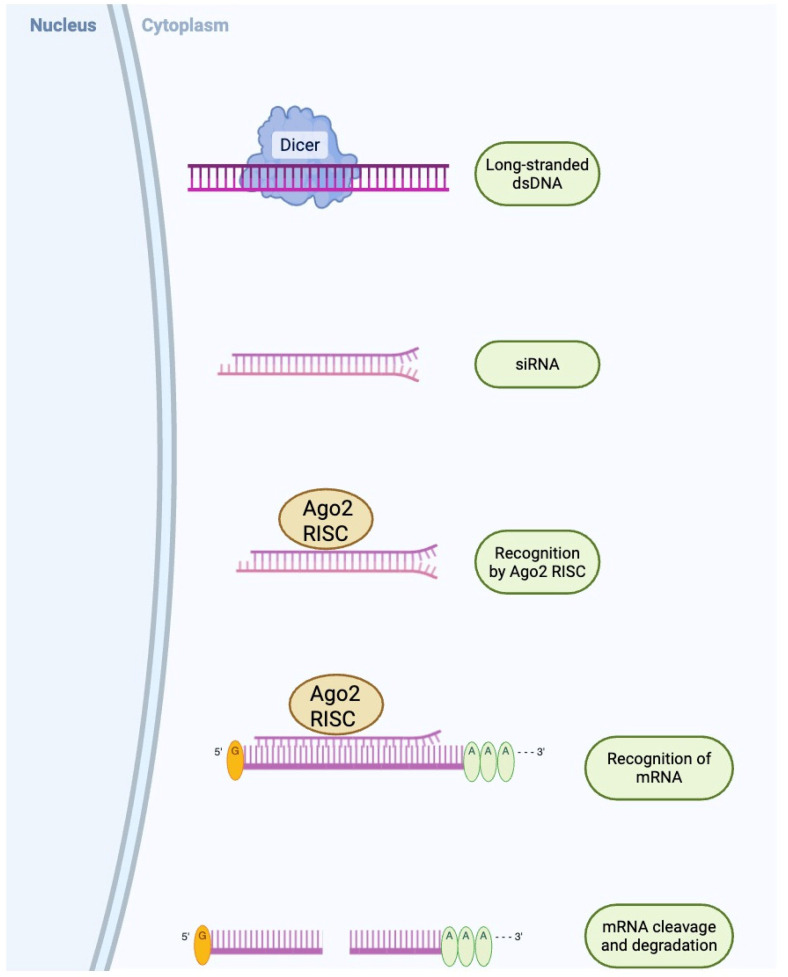
The process of RNA interference in eukaryotes. The siRNA pathway constitutes of double-stranded RNA (dsRNA) being cleaved by the dicer enzyme into siRNA, which are then recognized by the Ago-RISC, where one strand is then degraded and the other strand guides the complex to degrade complementary mRNA sequences [45]. Abbreviations: dsRNA, double-stranded ribonucleic acid; mRNA, messenger ribonucleic acid; Ago2, Argonaute 2; RISC, RNA-induced silencing complex.

In order to bind and destroy complementary mRNA targets and hence silence gene function, siRNA (composed of duplexes of about 21–23 base pairs that are integrated into the RNA-induced silencing complex (RISC)) have been utilized [45]. Once incorporated into RISC, the siRNA guide strand directs the complex to its complementary mRNA sequence, leading to precise cleavage and degradation of the target transcript, thereby preventing translation of the associated protein (Figure 2) [45]. In models of diabetic mice, siRNAs using LNPs that target oxidative stressors or pro-inflammatory cytokines such as tumor necrosis factor alpha (TNF-α) have shown improved wound healing outcomes and decreased systemic inflammation in shorter amounts of time compared to controls [46]. These results underscore the potential for RNA interference to produce rapid functional gains that can complement longer-lasting gene therapies. RNA interference tools targeting metabolic regulators such as protein tyrosine phosphatase 1B (PTP1B) and T-cell protein tyrosine phosphatase (TCPTP) have shown measurable insulin-sensitizing effects. Hydrodynamic injection of PTP1B short hairpin RNA (shRNA) in diabetic rodents suppressed PTP1B, enhanced hepatic protein kinase B phosphorylation, and lowered fasting glucose for five days following a single dose [47]. A parallel approach combining PTP1B and TCPTP siRNAs did not significantly improve efficacy, suggesting PTP1B alone is a primary target [47]. However, hydrodynamic delivery is impractical for humans. Recent advancements include exosome-based delivery of PTP1B siRNA, offering improved stability and tissue targeting with reduced toxicity [48]. Modified RNA-targeting sequence drugs targeting PTP1B have reached Phase II clinical trials, where they showed better insulin sensitivity and lower hemoglobin A1c (HbA1c levels) [48]. However, more testing is still needed. The majority of siRNA therapies for diabetes are still in the preclinical stage, but they highlight how effective RNA interference is at silencing harmful genes in metabolic diseases.

Combining gene silencing (e.g., siRNA against gluconeogenic enzymes) with gene addition (e.g., insulin transgenes) in dual-vector systems has shown synergistic effects on lowering fasting glucose levels in diabetic rodent models, emphasizing the potential of multi-modal gene therapy regimens for comprehensive metabolic correction [49]. Glucagon suppression, delayed gastric emptying, glucose-dependent insulin secretion, and extra-pancreatic effects on the central nervous system, cardiovascular system, kidneys, liver, and adipose tissues are some of the ways that GLP-1 receptor agonists enhance glycemic control [50]. A summary of all the findings from this section and Section 5 can be found in Table 3.

**Table 2:** Summary of the main therapeutic treatments and studies on rodents, dogs, and humans.

Species	Therapy	Outcome
NOD mice	Tolerogenic glutamic acid decarboxylase 65 (GAD 65) autoantigen mRNA vaccine	Prevention/delay of T1D onset; improved glucose tolerance [42].
Diabetic mice	Interleukin (IL-4) mRNA to enhance wound healing	Accelerated wound closure, reduced inflammation in diabetic mice [41].
STZ diabetic rats	GLP-1 gene therapy	Lower blood glucose, better insulin sensitivity & glucose tolerance, β-cell regeneration [24].
Autoimmune & chemically induced diabetic mice	PDX1 + MAFA to reprogram α-cells to insulin-producing cells	α → β-like cell reprogramming and correction of hyperglycemia [51].
Dogs	Insulin + Glucokinase via AAV1 in skeletal muscle	Normoglycemia, improved weight, normalized fructosamine, no hypoglycemia during exercise, sustained survival [25].
Adult with T2D	PTP1B inhibition (gene-silencing class)	Improved insulin sensitivity and HbA1c reduction [52].

Abbreviations: NOD, Non-obese diabetes; STZ, Streptozotocin; AAV1, Adeno-Associated Virus serotype 1; α-cell, Alpha Cell; β-cell, Beta Cell; GAD 65, Glutamic Acid Decarboxylase 65 kDa Isoform; GLP-1, Glucagon-Like Peptide 1; HbA1c, Hemoglobin A1c; *IL-4*, Interleukin 4; mRNA, Messenger Ribonucleic Acid; *MAFA*, Musculoaponeurotic Fibrosarcoma Oncogene Homolog A; *PDX1*, Pancreatic and Duodenal Homeobox 1; PTP1B, Protein Tyrosine Phosphatase 1B; T1D, Type 1 Diabetes; T2D, Type 2 Diabetes.

**Table 3:** An overview of the main gene and RNA-based approaches being explored to treat diabetes. It highlights how each method works, what results have been achieved so far, and the main challenges that remain.

Therapeutic Strategy	Mechanism/Target	Applications & Outcomes	Limitations/Disadvantages
Gene Addition (e.g., AAV1, AAV8 vectors)	Delivery of insulin, glucokinase, or transcription factors (PDX1, MAFA) to non-dividing cells.	Achieved long-term normoglycemia in diabetic dogs (8 years); α-cell reprogramming restored insulin production in rats.	Immunogenicity risk, limited cargo capacity, insertional mutagenesis (rare), potential for antibody-mediated inactivation.
Gene Editing (CRISPR/Cas9, Base & Prime Editing)	Correction of insulin receptor, PDX1, or β-cell regulatory loci.	Restores native glucose responsiveness without exogenous transgenes.	Off-target mutations, low HDR efficiency in quiescent β-cells, unpredictable genome alterations, ethical concerns.
Gene Silencing (siRNA, shRNA)	Knockdown of PTP1B, TXNIP, or other insulin resistance/apoptosis mediators.	Improved β-cell viability and reduced fasting glucose; synergy with insulin transgenes enhances control.	Transient effect, delivery inefficiency, potential hepatotoxicity, immune response if viral vector used.
Non-Viral & Hybrid Delivery (Lipid nanoparticles, electroporation, RNA complexes)	Non-integrative transfer of DNA/RNA cargo; co-delivery of siRNA and mRNA (e.g., insulin mRNA + PTP1B siRNA).	Enhanced β-cell viability and insulin secretion in T2D models; allows repeat dosing with low immunogenicity.	Lower transduction efficiency, transient expression, manufacturing complexity.
Cell-Based Therapy (iPSC/hESC-derived β-like cells, encapsulation)	Stem cell differentiation and ex vivo gene correction; immune-evasive β-cell generation.	Functional glucose-responsive β-cells; teratoma-free populations via GP2 sorting; proof-of-concept for neonatal diabetes correction.	Risk of teratoma from undifferentiated cells, alloimmune rejection, limited long-term graft survival, ethical issues.

Abbreviations: AAV1/8, (adeno-associated virus serotype 1/8); CRISPR, clustered regularly interspaced short palindromic repeats; DNA, deoxyribonucleic acid; HDR, high-dose rate; hESC, human embryonic stem cells; iPSC, induced pluripotent stem cells; *MAFA*, musculoaponeurotic fibrosarcoma oncogene family transcription factor A; mRNA, messenger ribonucleic acid; *PDX1*, Pancreatic and Duodenal Homeobox 1; PTP1B, protein tyrosine phosphatase 1B; RNA, ribonucleic acid; siRNA, small interfering ribonucleic acid; shRNA, short hairpin ribonucleic acid; GP2, glycoprotein 2; T2D, Type 2 Diabetes.

## 4. Current Strategies and Applications

AAV vectors are frequently used because of their ability to transduce non-dividing cells, although they still harbor some immunogenicity risk, whereas plasmid-based and other non-viral vectors have better safety records and far lower immunogenicity. For example, AAV1 vectors delivering insulin and glucokinase genes to skeletal muscle achieved long-term glycemic control in diabetic dogs over an eight-year period, demonstrating sustained transgene expression and metabolic correction without adverse effects (Table 2) [25]. *PDX1* and *MAFA* transcription factors have been precisely delivered to pancreatic α-cells using AAV8 vectors, reprogramming them into cells that produce insulin and restoring normal blood glucose levels in rats with diabetes (Table 2) [51]. Advantages of AAV vectors include their long-term expression and ability to transduce non-dividing cells, making them well-suited for treating chronic diseases such as diabetes, while some disadvantages are immunogenicity risks and limited cargo capacity, which can restrict the size of genes that can be delivered [53]. By contrast, non-viral methods, including lipid nanoparticles and electroporation, provide substitute delivery systems that can bypass both external and intracellular barriers with superior safety records; however, their transduction efficiencies tend to be lower than those of viral vectors [54,55]. Non-viral vectors also allow larger sizes, lower immunogenicity, and repeated administration if needed, but their expression is often transient, which may limit long-term therapeutic effects [56]. Emerging nanoparticle formulations co-delivering siRNA against PTP1B and mRNA encoding insulin have shown enhanced β-cell viability and function in T2D rodent models, suggesting a future for hybrid non-viral delivery systems in combinatorial gene therapy strategies [57]. Hybrid approaches combine the safety of non-viral vectors with the efficiency of RNA therapeutics, potentially overcoming delivery limitations, while AAV vectors provide robust glucose control [58]. Non-viral and hybrid strategies offer safer, versatile options for repeated or combinatorial therapies, emphasizing the need to tailor delivery to disease, safety, and treatment goals [59].

## 5. Challenges and Considerations

**Figure 3:**
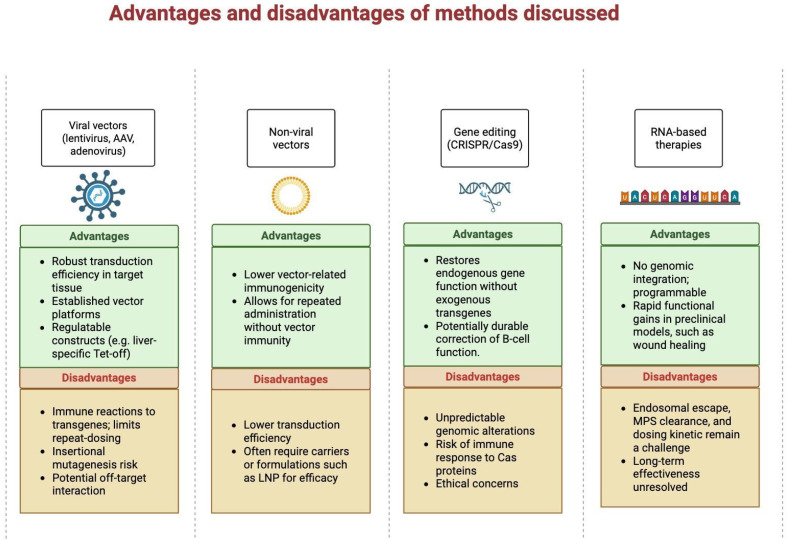
Advantages and limitations of principal delivery modalities and RNA therapeutics used in diabetes gene therapy. Each card summarizes primary benefits and key concerns for: viral vectors, non-viral systems, gene editing (CRISPR/Cas9), and RNA-based strategies (mRNA/siRNA with LNP delivery). This schematic highlights trade-offs between transduction efficiency, durability, immunogenicity and translational feasibility. Abbreviations: CAS (CRISPR-associated protein); MPS, (mononuclear phagocyte system).

Despite promising advancements, several challenges persist (Figure 3). Achieving tissue-specific gene delivery remains complex, requiring the development of vectors with precise targeting capabilities. The potential for immune reactions to viral vectors or introduced proteins creates a risk to therapy efficacy and patient safety [60]. Ensuring sustained and regulated expression of therapeutic genes is critical to avoid adverse effects like hypoglycemia. Additionally, the application of gene editing, especially in germline cells, raises ethical concerns and requires intensive regulatory monitoring [61].

Researchers in diabetes gene therapy generally coalesce around four major paradigms, each with distinct advantages and limitations. The first paradigm, gene addition, focuses on delivering functional insulin or insulin-sensitizing transgenes to compensate for β-cell failure or peripheral insulin resistance. It is likely that combining insulin and glucose-sensor enzymes would mimic key aspects of physiological regulation; however, muscle-based systems may not achieve the rapid, pulsatile insulin kinetics of native β-cells, posing a hypoglycemia risk if expression is not tightly controlled. For gene addition, integrating vectors (e.g., lentivirus) also carry insertional mutagenesis risks; even non-integrating AAVs can integrate at low frequency near oncogenes, as observed in animal studies [25]. Furthermore, capsid-specific antibodies may generate immune responses that limit repeated dosing and provoke islet-inflammation, jeopardizing long-term efficacy [31].

The second paradigm, gene editing, leverages CRISPR/Cas9 to correct disease-relevant loci or modulate endogenous gene networks. By precisely targeting insulin receptors, *PDX1* or other β-cell regulators, this approach promises restoration of native glucose responsiveness without exogenous transgenes. Critics of CRISPR emphasize ongoing challenges with off-target cleavage: high-fidelity Cas9 variants reduce but do not eliminate unintended cuts, and low homology-directed repair rates in quiescent β-cells limit correction efficiency [62]. In a large-scale in vivo investigation, Peterson et al. [63] performed whole-genome sequencing (WGS) on 50 CRISPR–Cas9-edited founder mice and 28 unedited controls to comprehensively assess genome-wide off-target activity across 163 guide RNAs. Their analysis identified a small but measurable number of single-nucleotide variants (SNVs) and small insertions/deletions (indels) occurring at non-targeted genomic sites, including some that were not predicted by existing in silico algorithms. Although the overall off-target mutation rate was relatively low, with only 8/163 guides (4.9%) exhibiting off-target activity, and comparable to background variation in controls, these findings emphasize that unanticipated genomic alterations can arise even under well-controlled editing conditions, which raises concerns for its application in humans. This underscores the importance of comprehensive WGS-based screening and improved predictive tools to ensure the safety and precision of in vivo CRISPR–Cas9 applications [63]. Strategies such as high-fidelity Cas9 variants and transient RNP delivery attenuate off-target activity but do not fully resolve it.

The third paradigm, gene silencing, targets pathogenic players in insulin resistance or β-cell apoptosis (e.g., PTP1B, TXNIP) using siRNA or shRNA. Early data demonstrate enhanced β-cell viability and reduced fasting glucose in rodent models, suggesting synergy between addition and silencing modalities [64]. However, single-agent silencing alone does not replace lost β-cell mass, and durable delivery of RNAi constructs remains problematic without invoking immunogenic viral vectors [65,66]. Additionally, long-term expression of shRNAs has elicited hepatotoxicity in preclinical models due to saturation of the endogenous miRNA machinery [64].

Finally, cell-based gene therapy involves generating autologous iPSC-derived β-like cells or encapsulated islet grafts engineered for immune evasion. CRISPR/Cas9-edited stem cells to correct monogenic mutations have provided proof of concept for ex vivo gene correction in neonatal diabetes [67]. While cell-replacement approaches offer the potential for permanent β-cell restoration, critics highlight hurdles in differentiation fidelity, transplantation site selection, and immune protection that must be resolved before broad clinical adoption [68,69]. In cell-based therapy, residual undifferentiated iPSCs can form teratomas unless rigorous purification and suicide-gene safeguards are employed; for example, a paper demonstrated that purifying hESC-derived pancreatic progenitors based on the surface marker glycoprotein 2 (GP2) completely eliminates teratoma risk and yields functional, glucose-responsive β cells after transplantation. To prove this, researchers separated three populations of pancreatic progenators (PPs) using magnetic-activated cell sorting (MACS), with the populations being derived from an hESC-H1 line that constantly expresses DsRed fluorescent protein (RFP-H1) and then transplanted them into immunocompromised mice: non-sorted (bulk population), flow-through (negative for glycoprotein (GP2)), and GP2^+^ (purified pancreatic progenitors). Their results showed teratoma/outgrowth rates of non-sorted (NS) 27% (4/15), FT 18% (3/17) and 0% (0/11) in GP2-enriched pancreatic progenitors, demonstrating that targeted purification has the potential to eliminate teratoma formation [70]. Even fully differentiated β-like cells risk alloimmune rejection without encapsulation or immunomodulation. Martin et al. (2020) inserted an inducible iCasp9 suicide switch into the NANOG (pluripotency-specific) locus of hPSCs, enabling selective ablation of undifferentiated cells and preventing teratoma formation in vivo [71]. To do this, they developed a dual fail-safe system in hESC-derived β-cell progenitors by coupling lineage-restricted expression with a pro-apoptotic (iCasp9) payload and it abolished tumorigenicity in their murine models., which eliminated residual pluripotent cells and abolished tumorigenicity in murine transplantation models [71]. Even fully differentiated hPSC-derived β-like cells remain susceptible to alloimmune rejection. For example, a study demonstrated that hESC-derived pancreatic progenitors elicit adaptive immune responses in hosts, which may be overcome by transplanting more cells or choosing an alternative site of transplantation, despite induced immunosuppression following the transplant [72]. Additionally, Wang et al. (2021) describe further local immunomodulatory strategies, such as co-transplation with regulatory T-cells or mesynchmal stromal cells, that can reduce allo-graft rejection and improve graft survival [73]. Collectively, these findings underscore that both robust negative selection of undifferentiated cells via suicide switches and local immunomodulatory strategies, such as encapsulation or co-transplantation with regulatory T cells, are critical to ensure the safety and longevity of stem cell–based β-cell therapies. See Table 3 for a consolidated summary of the results discussed in this section and Section 3.

## 6. Efficacy Considerations

Debate also centers on durability and physiological regulation of therapeutic effects. The muscle-directed insulin/glucokinase system maintained normoglycemia for eight years in dogs, but human translation requires adapting vector doses to larger muscle mass and verifying dynamic insulin response under variable metabolic loads [25,64]. CRISPR correction of insulin receptor loci offer tighter glucose sensing but struggles with low Homology-Directed Repair (HDR) efficiency in post-mitotic β-cells, limiting long-term correction in large-animal or humanized models [31,62]. Gene silencing (e.g., PTP1B knockdown) improves insulin sensitivity transiently but does not address β-cell loss; combinations with insulin transgenes in dual-vector regimens show promise in rodents, lowering fasting glucose more effectively than monotherapy [64,74]. Cell-based approaches have produced glucose-responsive β-like cells in vitro, but post-transplant survival and function decline over time due to hypoxia and immune attack, requiring further engineering for vascularization and immune evasion [62].

To further improve efficacy, strategies could include optimizing vector targeting and delivery efficiency, adjusting dosing or repeating administrations to maintain therapeutic effects, and using inducible or glucose-responsive promoters to better mimic natural insulin regulation. Enhancing HDR efficiency or applying base/prime editing in β-cells may allow more lasting gene correction, while combination therapies addressing multiple disease mechanisms simultaneously could further improve outcomes. For cell-based therapies, engineering cells for improved vascularization, resistance to hypoxia, and immune evasion can help maintain their function in vivo [75].

## 7. Ethical Debates

Ethical controversies cluster around germline versus somatic editing, access and equity, and enhancement concerns. Nearly all stakeholders agree that germline editing for diabetes is unjustifiable given unpredictable multigenerational risks; major bodies (e.g., World Health Organization (WHO), National Academy of Sciences (NAS)) prohibit heritable modifications until safety is assured [31,62]. In somatic editing, debates weigh life-altering benefits for patients with brittle T1D against risks of off-target mutations and immune reactions. Ethicists argue that gene therapy aligns with beneficence and justice if informed consent, independent oversight, and post-trial monitoring are guaranteed [74,76]. Critics counter that the high costs of advanced gene- and cell-based therapies—often projected in the $100 K–$500 K range per patient—could exacerbate healthcare disparities by restricting access to only those who can afford them. An Multidisciplinary Digital Publishing Institute (MDPI) review notes that “fair access to advanced treatments like gene therapy poses a significant challenge, as the high costs associated with these treatments could exacerbate health disparities, making them accessible only to those who can afford them and leaving underserved populations without these potentially life-changing interventions” [77].

Reconciling these divergent schools and controversies demands multidisciplinary frameworks that integrate robust preclinical data, transparent ethical review, global regulatory alignment, and patient-focused outcomes. Only through collaborative efforts can gene therapy for diabetes evolve safely, effectively, and equitably from experimental promise to clinical reality.

## 8. Conclusions

Gene therapy and RNA-based therapeutic strategies are transforming diabetes treatment by targeting the disease at its genetic and molecular roots, offering innovative alternatives to traditional approaches. This review examined the mechanistic and clinical potential of these therapies, from gene addition, editing and silencing to RNA-based modalities like mRNA and siRNA. Advances in delivery vectors, both viral and non-viral, have enabled tissue-specific targeting and safer, more efficient therapeutic expression, while hybrid strategies, such as co-delivering insulin mRNA and PTP1B siRNA, offer promising synergies that combine baseline correction with real-time metabolic tuning. RNA therapies have shown particular promise in reducing inflammation, modulating immune responses, and improving insulin sensitivity, with mRNA-based agents already advancing toward clinical trials. To realize this potential, rigorous translational studies are needed that jointly optimize therapeutic design, delivery logistics, and long-term safety monitoring. However, the field faces significant challenges, including the need for precise regulatory control, minimization of off-target effects, and long-term durability. Ethical concerns, such as equitable access, informed consent, and the cost burden of these therapies, remain critical, especially as global health disparities threaten to limit access in low- and middle-income regions. Nonetheless, the continued evolution of high-fidelity gene editing tools, advanced delivery methods, and regulatory frameworks pave the way for transformative progress. Ultimately, the integration of gene and RNA therapies into mainstream diabetes care depends on multidisciplinary innovation and global cooperation, and future studies will be essential to refine these approaches, validate long-term safety, and ensure their translation into equitable, personalized, and sustainable solutions for diabetes worldwide.

## Figures and Tables

**Table 1:** A comparison of the characteristics of type 1 and type 2 diabetes.

Type 1 Diabetes	Type 2 Diabetes
Autoimmune destruction of beta cells of the pancreas	Insulin resistance (in muscle, fat, liver)
↓ insulin production → absolute insulin deficiency	↓ cellular response to insulin → relative deficiency
Rapid onset, usually in young	Gradual onset, usually adults
Requires insulin therapy	Lifestyle ± oral meds ± insulin therapy

## Data Availability

No new data were generated in the writing of this review.
